# Nematicidal Activity of Grammicin Biosynthesis Pathway Intermediates in *Xylaria grammica* KCTC 13121BP against *Meloidogyne incognita*

**DOI:** 10.3390/molecules26154675

**Published:** 2021-08-02

**Authors:** Yoon Jee Kim, Kalaiselvi Duraisamy, Min-Hye Jeong, Sook-Young Park, Soonok Kim, Yookyung Lee, Van Thi Nguyen, Nan Hee Yu, Ae Ran Park, Jin-Cheol Kim

**Affiliations:** 1 Department of Agricultural Chemistry, Institute of Environmentally Friendly Agriculture, College of Agriculture and Life Science, Chonnam National University, Gwangju 61186, Korea; khdg45@naver.com (Y.J.K.); dkalaiselvi19@gmail.com (K.D.); yousy68@naver.com (Y.L.); vanbafu91@gmail.com (V.T.N.); nanheeyu0707@gmail.com (N.H.Y.); arpark9@naver.com (A.R.P.); 2 Department of Plant Medicine, Sunchon National University, Suncheon 57922, Korea; minhye1962@gmail.com (M.-H.J.); spark@scnu.ac.kr (S.-Y.P.); 3 Biological and Genetic Resources Assessment Division, National Institute of Biological Resources, 42 Hwangyeong-ro, Incheon 22689, Korea; sokim90@korea.kr

**Keywords:** biosynthesis intermediates, grammicin, *Meloidogyne incognita*, nematicidal activity, *Xylaria grammica*

## Abstract

Grammicin, a polyketide metabolite produced by the endolichenic fungus *Xylaria grammica* KCTC 13121BP, shows strong nematicidal activity against *Meloidogyne incognita.* This study was performed to elucidate the grammicin biosynthesis pathway of *X. grammica* KCTC 13121BP and to examine the nematicidal activity of the biosynthesis intermediates and derivatives against *M. incognita*. Two grammicin biosynthesis intermediates were isolated from a T-DNA insertion transformant (strain TR-74) of *X. grammica* KCTC 13121BP and identified as 2-(hydroxymethyl)cyclohexa-2,5-diene-1,4-dione (compound **1**) and 2,5-dihydroxybenzaldehyde (compound **2**), which were also reported to be intermediates in the biosynthesis pathway of patulin, an isomer of grammicin. This indicates that the grammicin biosynthesis pathway overlaps almost with that of patulin, except for the last few steps. Among 13 grammicin biosynthesis intermediates and their derivatives (except grammicin), toluquinol caused the highest *M. incognita* J2 mortality, with an LC_50/72 h_ value of 11.13 µg/mL, which is similar to grammicin with an LC_50/72 h_ value of 15.95 µg/mL. In tomato pot experiments, the wettable powder type formulations (WP) of toluquinol (17.78 µg/mL) and grammicin (17.78 µg/mL) also effectively reduced gall formation on the roots of tomato plants with control values of 72.22% and 77.76%, respectively, which are much higher than abamectin (16.67%), but lower than fosthiazate (100%). The results suggest that toluquinol can be used directly as a biochemical nematicide or as a lead molecule for the development of new synthetic nematicides for the control of root-knot nematode diseases.

## 1. Introduction

Root-knot nematodes (RKNs) cause great harm to global agricultural productivity, reducing crop yields by 12–14% and causing global economic losses amounting to more than $125 billion—annually [1,2]. The second-stage juveniles (J2) of RKNs attack roots, inducing the formation of giant cells after infection and disturbing plant hormone production. J2 also induces abnormal cell division, thus generating root galls. Infection by RKNs reduces water and nutrient absorption by hampering plant root systems, resulting in hindered plant growth and subsequent disease complexes [3]. Although chemical nematicides are the primary method of controlling nematode infections, the use of some of them is prohibited in several countries because of their negative impact on human health and the environment [4,5].

Currently, biological control methods using microorganisms (such as bacteria and fungi) and their metabolic products are being actively studied. Endophytic fungi are microorganisms that live below the epidermal cell layer in plant tissues without causing any symptoms of disease. They form symbiotic relationships with their host plants, producing secondary metabolites that protect against pathogen attacks [6,7]. Substances produced by endophytic fungi have a variety of effects on plants, including growth promotion, increased resistance to disease, and an enhanced ability to withstand environmental stress [8]. Therefore, such metabolites have the potential to be used as biological control agents. Secondary metabolites of endophytic fungi include alkaloids, phenols, steroids, terpenoids, quinones, peptides, polyketides, and acids [9]. Polyketides are natural substances produced by the continuous condensation of acetate units. Polyketide compounds include antibiotics, antifungals, anticancer agents, immunosuppressants, and anti-dyslipidemia agents. They are clinically useful, and form the biologically active components of various substances used as agricultural growth-promoters, pesticides, and anthelmintics [10]. Among them, amphotericin B (produced by *Streptomyces nodosus* Trejo) is an essential antifungal medication in humans. Amphotericin B also displays biological action against some viruses, protozoans, and prions via sterol interaction [11]. Rifamycin S, synthesized by *Amycolatopsis rifamycinica* has been used for the treatment of a variety of diseases, particularly HIV-related tuberculosis. Despite their structural diversity, polyketides are synthesized by a common biosynthesis mechanism [12].

Previously, we reported the nematicidal effect of a polyketide metabolite, grammicin, produced by the endolichenic fungus *Xylaria grammica* KCTC 13121BP [13]. Grammicin is a stereo isomer of patulin, which is one of the mycotoxins produced by various fungal species, such as *Penicillium* spp., *Aspergillus* spp., and *Byssochlamys* spp. [14]. Even though the patulin biosynthesis pathway has been elucidated well [15,16], the study of the biosynthesis pathway of grammicin has not been undertaken. During the study on the elucidation of the biosynthetic pathway in the *X. grammica* KCTC 13121BP, we found that two substances isolated from *X. grammica* KCTC 13121BP transformant are patulin biosynthesis intermediates and toxic to the second-stage juveniles of *Meloidogyne incognita*, a causal agent of root-knot nematode diseases. Therefore, the objectives of this study were: (1) to isolate and identify grammicin biosynthesis intermediates from the culture filtrate of *X. grammica* KCTC 13121BP transformant and evaluate their in vitro nematicidal activity against *M. incognita* and *Caenorhabditis elegans*, (2) to examine the in vitro nematicidal activity of various grammicin biosynthesis intermediates and their derivatives, and (3) to evaluate the disease control efficacy of several substances against *M. incognita* in tomato.

## 2. Results and Discussion

*Meloidogyne incognita* is a dominant plant parasitic RKN that causes severe damage to several agriculture crops worldwide. Since *M. incognita* is polyphagous, its complete eradication from soils is challenging. Although chemical nematicides are the primary means of managing nematodes, biological control is a safe alternative [17,18]. The culture filtrate of the endolichenic fungus *X. grammica* is reported to have satisfactory nematicidal activity, while the active compound from *X. grammica* KCTC 13121BP, grammicin, shows strong nematicidal activity against *M. incognita* [13]. Even if a number of compounds from fungi are reported as suitable for biological control, difficulties remain in culture techniques [19]. In this study, we aimed to illustrate the biosynthesis pathway of grammicin. A total of 2500 TRs of *X. grammica* KCTC 13121BP were screened using ATMT (Agrobacterium Tumefaciens-Mediated Transfer) to find TRs (Transformants) that do not synthesize grammicin by random mutagenesis. From TLC and HPLC analyses, we confirmed that 16 TRs did not synthesize grammicin. Interestingly, three strains (TR-67, TR-74, and TR-94) showed reduced grammicin production. Furthermore, TLC analysis found that they produced two quenching compounds.

**Figure 1:**
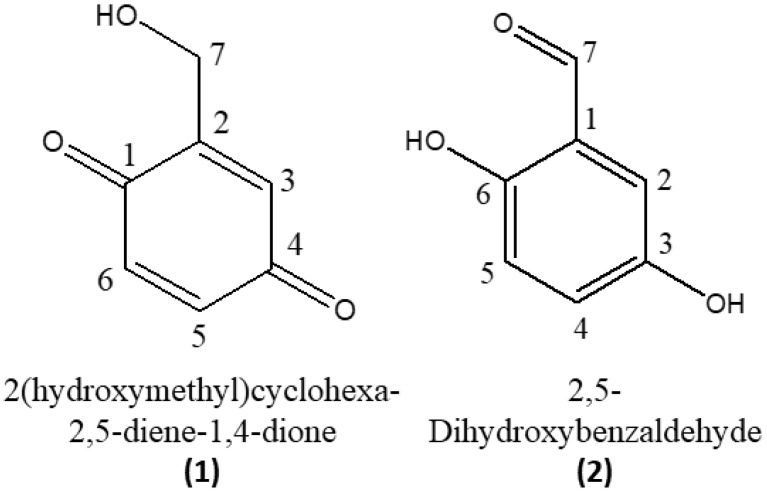
Chemical structure of compounds **1** and **2**, isolated from the culture filtrate of the grammicin-knockout mutant strain (TR74) of *Xylaria grammica*.

To isolate the two compounds produced by TR-67, TR-74, and TR-94 of *X. grammica* KCTC 13121BP, TR-74 culture filtrate was extracted with EtOAc and then purified by silica gel column chromatography with a Sep-Pak C18 cartridge. The EI mass spectra of compounds **1** and **2** displayed a molecular ion at *m*/*z* 138 [M]^+^. Based on the EI mass and NMR spectral data, the molecular formulas of the two compounds were determined to be C_7_H_6_O_3_. By interpreting all EI mass and NMR spectral data, compound **1** was identified as 2-(hydroxymethyl)cyclohexa-2,5-diene-1,4-dione and compound **2** as 2,5-dihydroxybenzaldehyde (Figure 1). The NMR data for compounds **1** and **2** are summarised in Supplementary Tables S1 and S2, respectively. Compound **1**, also known as gentisinquinone, is produced by the mycelial fungi *Penicillium patulum* and *P. urticae*, as well as the marine-derived fungus *Phoma* sp. FOM-8108. Gentisinquinone has been reported for its antibiotic, herbicide, and sphingomyelinase inhibitor capabilities [20]. Compound **2**, also known as gentisaldehyde, is known to be an antimicrobial agent against *Staphylococcus aureus*, which causes bovine mastitis. The MIC_50_ of gentisaldehyde against *S. aureus* is 500 mg/L and 67% of *S. aureus* isolates show an MIC of 500 mg/L for gentisaldehyde [21]. Additionally, gentisaldehyde has been used against *Campylobacter jejuni*, *Escherichia coli*, *Listeria monocytogenes*, and *Salmonella enterica* [22,23].

**Figure 2:**
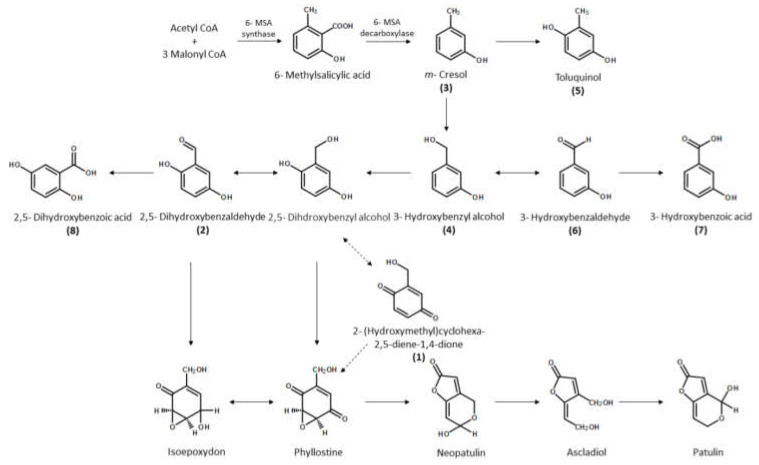
Biosynthesis of grammicin, an isomer of patulin, and its intermediates used in this study (adapted from [16]).

Compounds **1** and **2** are known to be intermediates in the biosynthesis pathway of patulin, an isomer of grammicin. Thus, grammicin is synthesized via a similar biosynthetic pathway (Figure 2). *Cis*-jasmone is synthesized from such biosynthetic pathways, using *iso*-12-oxophytodienoic acid (*iso*-OPDA, an isomer of *cis*-OPDA) as an early precursor. Our two compounds share some elements of their biosynthetic pathways and synthesize *Cis*-jasmone in a similar way [24]. The synthesis of patulin begins with the formation of 6-methylsalicylic acid via synthesis of a single acetyl-CoA and three malonyl-CoA units [16].

**Figure 3:**
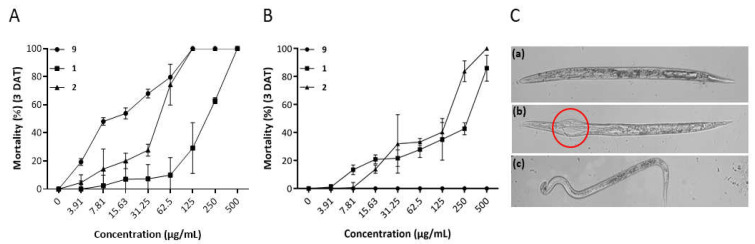
In vitro nematicidal activity of grammicin (**9**), compound **1,** and compound **2** against second-stage juveniles of *Meloidogyne incognita* (**A**), fourth larval stage (L4) worms of *Caenorhabditis elegans* (**B**), and morphological variations in *M. incognita* (**C**) treated with grammicin (a), compound **2** (b), and control (c). Each value represents the means ± standard deviation with three replicates against *M. incognita*. Relationships among means were analysed with one-way analysis of variance and Duncan’s multiple-range test (*p <* 0.05).

The mortality percentage of *M. incognita* exposed to various concentrations of grammicin, compound **1**, and compound **2** for 72 h are shown in Figure 3A. The J2 mortality increased as the concentration of the test compounds increased. Grammicin and compound **2** had significantly higher (*p* < 0.05) nematicidal activity towards *M. incognita* than compound **1**, exhibiting LC_50/72 h_ values of 15.95 and 42.43 µg/mL, respectively (Table 1). However, in vitro, compounds **1** and **2** showed lower nematicidal activity against *M. incognita* J2 than grammicin. Furthermore, results from the free-living *C*. *elegans* in vitro study were at odds with those of the *M. incognita* studies; here, compounds **1** and **2** induced significant (*p* < 0.05) mortality percentages, whereas grammicin did not induce mortality up to 500 µg/mL (Figure 3B). Microscopic observation indicated that compound **2** developed an anomalous feature in dead *M. incognita* (Figure 3C). This remarkable morphological modification is potentially related to compound **2′**s entry into nematodes and its interference in vital processes related to the oesophageal glands and nerve ring; this should be investigated further. These data indicate that the mechanisms of nematicidal action may differ between grammicin and compounds **1** and **2** (or between all three). To the best of our knowledge, this is the first study on the nematicidal activity of compounds **1** and **2** against *M. incognita* and *C. elegans*.

**Figure 4:**
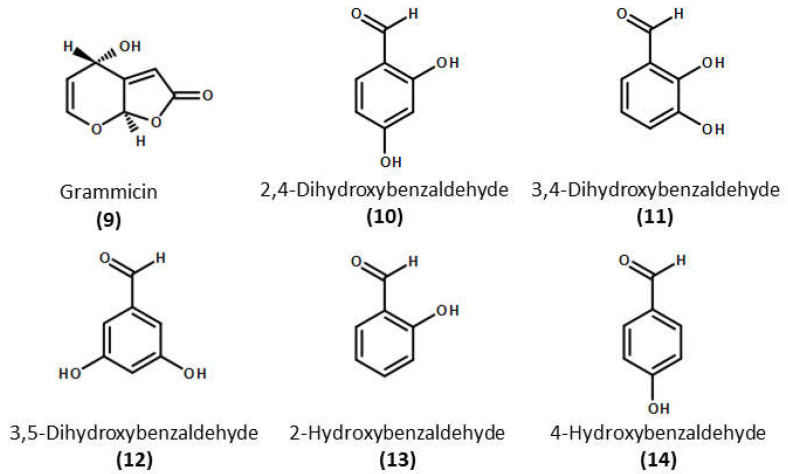
Chemical structure of the hydroxybenzene derivatives (compounds **9**–**14**) used in this study.

**Figure 5:**
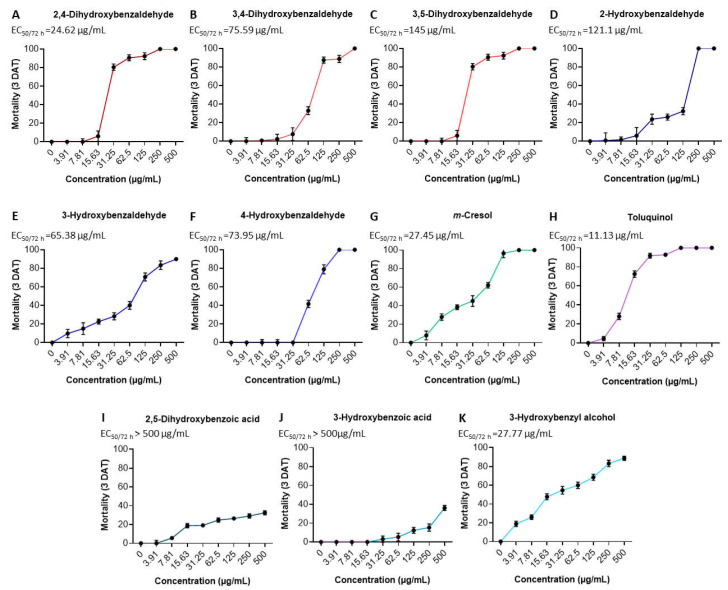
Effect of 2,4-dihydroxybenzaldehyde (**A**), 3,4-dihydroxybenzaldehyde (**B**), 3,5-dihydroxybenzaldehyde (**C**), 2-hydroxybenzaldehyde (**D**), 3-hydroxybenzaldehyde (**E**), 4-hydroxybenzaldehyde (**F**), m-cresol (**G**), toluquinol (**H**), 2,5-dihydroxybenzoic acid (**I**), 3-hydroxybenzoic acid (**J**), and 3-hydroxybenzyl alcohol (**K**) on J2 of Meloidogyne incognita three days after treatment. Each value represents the mean ± standard deviation with three replicates. DAT = days after treatment.

In order to discover new natural metabolites that show strong nematicidal activity against *M. incognita*, the nematicidal activity of the other two grammicin biosynthesis pathway intermediates (**3** and **4**, Figure 2), their by-products (**5**–**8**, Figure 2), and derivatives (**10**–**14**, Figure 4) were tested against J2 of *M. incognita* and compared with grammicin. Significantly (*p* < 0.05), toluquinol (**5**), 2,4-dihydroxybenzaldehyde (**10**), *m*-cresol (**3**), and 3-hydroxybenzyl alcohol (**4**) were the most effective against J2 of *M. incognita*, with LC_50/72 h_ values of 11.13, 24.62, 27.45, and 27.77 µg/mL, respectively. This was followed by 3-hydroxybenzaldehyde (**7**), 4-hydroxybenzaldehyde (**14**), 3,4-dihydroxybenzaldehyde (**11**), and 2-hydroxybenzaldehyde (**13**), with LC_50/72 h_ values of 65.38, 73.95, 75.59, and 121.1 µg/mL, respectively (Table 1; Figure 5). Among the 14 compounds tested in this study, only toluquinol (**5**) resulted in a higher J2 mortality in *M. incognita* than that of grammicin (**9**). The LC_50/72 h_ values of 2,5-dihydroxybenzoic acid (**8**) and 3-hydroxybenzoic acid (**7**) were above 500 µg/mL. Mahajan et al. (1992) [25] demonstrated that *M. incognita* treated with either 1,100 µg/mL 2,5-dihydroxybenzoic acid (**8**) or 3-hydroxybenzoic acid (**7**) showed mortality rates of 75.5 and 54.9%, respectively, after 24 h exposure. When the compounds were tested against J2 of *M. incognita*, mortality responses were clearly dose dependent. Our findings are similar to those of Cheng et al., (2017) [26], who reported that the mortality percentage is directly related to compound concentration. A previous study also reported that 6-methylsalicylic acid and *m*-cresol, the first and second precursors to polyketide-derived epoxycyclohexenol, exhibited a strong inhibitory effect on *M. incognita* and *C. elegans* [27].

As for structure-activity relationships, out of the three 3-hydroxybenzyl chemicals (**4**, **6**, and **7**), compound **4** has an alcohol group and was the most active. This was followed by compound **6**, which has an aldehyde group, and **7**, which has a carboxylic acid group. The 2,5-dihydroxybenzyl compounds (**1**, **2**, **5**, and **8**) and toluquinol (**5**), which has a methyl group, displayed the strongest activity. This was followed by compound **2**, which has an aldehyde group. The remaining two compounds (**1**, with an alcohol group, and **8**, with a carboxylic acid group) were virtually inactive. Of the three dihydroxybenzaldehyde chemicals (**10**, **11**, and **12**), compound **10** was the most active, followed by **11** and **12**. In addition, the hydroxybenzaldehyde with a hydroxy group in ortho-position (**13**) was less toxic compared with that of chemicals with hydroxy groups in other positions (**6** and **14**). Caboni et al. (2013) [28] also reported that 2-hydroxybenzaldehyde was the most effective paralytic compound against *M. incognita*, followed by 3-hydroxybenzaldehyde and 4-hydroxybenzaldehyde. Overall, the benzene chemicals containing a methyl group, such as compounds **3** and **5**, resulted in high J2 mortality in *M. incognita*, whereas the benzene chemicals containing carboxylic acid, such as compounds **6** and **8**, were virtually inactive. This information may be useful for the development of new synthetic nematicides, based on a hydroxybenzene skeleton for the control of root-knot nematode diseases.

**Figure 6:**
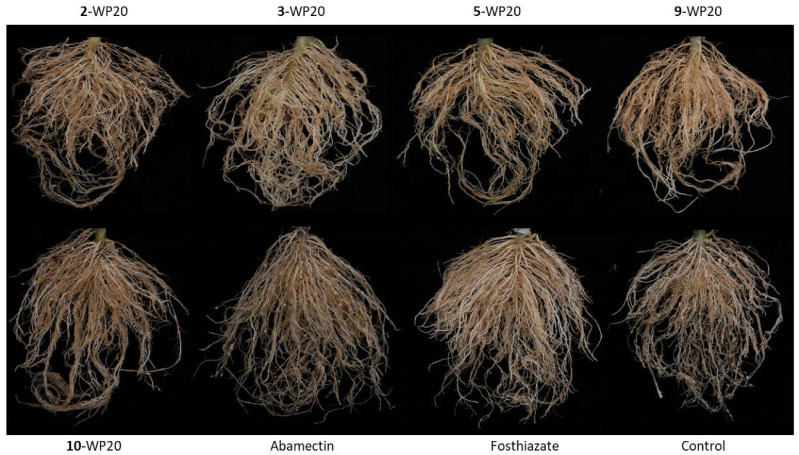
Effect of wettable powder formulations of 2,5-dihydroxybenzaldehyde (2-WP20), m-cresol (3-WP20), toluquinol (5-WP20), grammicin (9-WP20), and 2,4-dihydroxybenzaldehyde (10-WP20) on gall formation in tomato roots infested with *Meloidogyne incognita*. Fosthiazate (4000-fold dilution) and abamectin (5000-fold dilution) were used as positive controls.

Based on the in vitro nematicidal activity, the disease control efficacy of compounds **2**, **3**, **5**, **9**, and **10** was assessed for tomato root-knot nematode diseases in pot experiments. WP20 samples of compounds **5** and **9** were the most effective at suppressing the formation of galls on tomato roots by 72.22% and 77.78%, respectively (Table 2). On untreated tomato roots, numerous large root galls were found, whereas smaller and fewer galls were found on tomato roots treated with **5**- or **9**-WP20 (Figure 6). The disease control efficacy of these two formulations was significantly higher than that of abamectin and comparable with that of fosthiazate. 

In summary, this study indicates that grammicin is synthesized via a similar biosynthesis pathway to patulin and that grammicin biosynthesis intermediates, by-products, and derivatives have moderate to strong nematicidal activity against J2 of *M. incognita*. Additionally, toluquinol has more or less similar in vitro and in vivo nematicidal activity to that of grammicin. In particular, its WP20 formulation displayed greater disease control efficacy on tomato plants than the positive control, abamectin. Additionally, no phytotoxic symptoms were observed for toluquinol (**5**-WP20) treatment (Table 2). A previous study revealed that toluquinol isolated from fungi in the soil has herbicidal activity [29], while toluquinol isolated from a marine fungus, *Penicillium* sp., has shown antitumour, antiangiogenic, and antilymphangiogenic properties. Toluquinol treatment inhibits the proliferation of tumour cells, such as HL60 leukaemia, HT1080 fibrosarcoma, and HT29 colon adenocarcinoma cells, with IC_50_ values of 3.2, 8.6, and 5.8 μM, respectively. Toluquinol has also been shown to inhibit the proliferation of cultured bovine aortic endothelial cells, with an IC_50_ value of 2.3 μM, and it shows maximum angiogenesis inhibition in chicken, mouse, and zebrafish models at concentrations of 20, 30, and 20 nM, respectively [30]. Furthermore, toluquinol suppresses lymphangiogenesis by down-regulating the VEGF-C/VEGFR-3 cascade [31]. To the best of our knowledge, this is the first study on nematicidal activity of toluquinol (**5**) against *M. incognita*. Since this compound has a very simple chemical structure without chiral carbon, it can be used as a lead molecule for the development of new synthetic nematicides. In addition, our comparative study on the nematicidal activity of various hydroxybenzene derivatives is believed to be of great help in the development of such nematicides. Toluquinol (**5**) may also be used directly as a natural nematicide for the control of RKN diseases (if its toxicity is not an issue) or as a lead molecule for the development of new synthetic nematicide.

## 3. Materials and Methods

### 3.1. Chemicals

Analytical standards of 2,5-dihydroxybenzaldehyde (compound **2**; 98% purity), *m*-cresol (**3**; 99% purity), 3-hydroxybenzyl alcohol (**4**; 99% purity), toluquinol (**5**; ≥98% purity), 3-hydroxybenzaldehyde (**6**; ≥99% purity), 3-hydroxybenzoic acid (**7**; 99% purity), 2,5-dihydroxybenzoic acid (**8**; 98% purity), 2,4-dihydroxybenzaldehyde (**10**; 98% purity), 3,4-dihydroxybenzaldehyde (**11**; 97% purity), 3,5-dihydroxybenzaldehyde (**12**; 98% purity), 2-hydroxybenzaldehyde (**13**; 98% purity), and 4-hydroxybenzaldehyde (**14**; 98% purity) were procured from Sigma-Aldrich, Seoul, Korea. Methanol, acetone, and water used for the following experiments were of high-performance liquid chromatography (HPLC) grade.

### 3.2. Fungal Strain and Nematode Culture

*X. grammica* KCTC 13121BP (isolated from the lichen *Menegazzia primaria*) was stored, as described previously [13]. The fungal strain was cultured on potato dextrose agar (PDA) (Becton, Dickinson and Co., Sparks, MD, USA) at 25 °C. The agar plugs containing fresh mycelia were inoculated into 50 mL of potato dextrose broth (PDB) in a 250 mL Erlenmeyer flask and then incubated on an orbital shaker at 25 °C and 150 rpm for 14 days to obtain culture filtrate for experiments.

The population of *M. incognita* was maintained on tomato plants (*Solanum lycopersicum* L. cv. Seokwang; Farm Hannong Co., Seoul, Korea) in a greenhouse at 25 ± 5 °C. Infected tomato plants were uprooted, and their roots were washed with running water to remove soil. Roots were then cut into 1 cm long pieces. Nematode eggs were extracted using 0.5% (*v*/*v*) sodium hypochlorite (NaOCl) [32] and allowed to hatch in modified Baermann funnels at 28 °C [33]. Synchronized first larval stage wild-type (Bristol N2) *Caenorhabditis elegans* were kindly provided by Prof. Junho Lee, Division of Biological Sciences, Seoul National University, South Korea. To obtain L4 worms, the L1 worms were spread on nematode growth media carrying a lawn of *E. coli* OP50 and incubated at 20 °C.

### 3.3. Agrobacterium Tumefaciens-Mediated Transfer (ATMT) of X. grammica KCTC 13121BP

We carried out ATMT, according to previously described procedures, with slight modification [34]. Briefly, *A*. *tumefaciens* AGL-1, harbouring the binary vector pSK1044, was used for random mutagenesis of *X. grammica* KCTC 13121BP. The vector pSK1004 contains the hygromycin B phosphotransferase and enhanced green fluorescence protein genes in the T-DNA region, acting as a selection marker in fungal transformants [34]. AGL-1 cells were predisposed by incubating 10% (*v*/*v*) of 2-day-old inoculum from minimal medium for 6 h in an induction medium containing 200 µM acetosyringone at 28 °C and 150 rpm. Fungal cells for transformation were prepared by grinding young mycelia grown in PDB and then mixing with equal volumes of predisposed *A. tumefaciens.* Subsequently, 200 µL of the mixture was spread on a sterile cellulose nitrate membrane filter (Whatman^TM^ NC45, GE Healthcare UK Limited, Buckinghamshire, UK) and placed on co-cultivation medium containing 200 µM acetosyringone. After co-cultivation, membranes were transferred to a selection medium: PDA supplemented with 50 µg/mL hygromycin B and 250 µg/mL cefatoxim. Approximately one week later, individual colonies growing on membrane filters were transferred to new PDA plates.

### 3.4. Assay of Grammicin Biosynthesis in Transformants

The culture filtrate of *X. grammica* KCTC 13121BP was extracted with an equal volume of ethyl acetate (EtOAc; 1:1, *v*/*v*) by sonication [13]. A portion of the EtOAc layer (10 µL) was applied to a thin-layer chromatography (TLC) plate (20 × 20 cm; Kiesel gel 60 F254, 0.2 mm thick; Merck, Darmstadt, Germany), which was developed using a solvent of CHCl_3_/MeOH (93:7, *v*/*v*). The grammicin spot was observed under ultraviolet (UV) light (254 nm). Transformants (TRs) with no, or reduced, synthesis of grammicin (compared to a wild-type strain) that produced other compounds were selected and incubated in PDB (50 mL) for 14 days at 25 °C and 150 rpm in the dark. Each culture filtrate was partitioned twice with equal volumes of EtOAc. Then, the organic solvent layer was concentrated to dryness in a rotary evaporator in vacuo. The residue was redissolved in 4 mL methanol. To analyse grammicin and the accumulated metabolites in the wild-type and selected TR extracts, the organic extracts were subjected to HPLC using a system equipped with a LC-20AT HPLC pump and SPD-M20A PDA detector (Shimadzu Corp., Kyoto, Japan). The HPLC column used was the C18 (XBridge, 5 µm, 4.6 × 250 mm; Waters Corp., Milford, MA, USA) and the mobile phase was 0.1% trifluoroacetic acid (TFA; 99% purity; Sigma-Aldrich, St. Louis, MO, USA) in water (eluent A) and 0.1% TFA in acetonitrile (eluent B). The gradient applied was linear from 0 to 40% B in 30 min and from 40 to 0% B in 2 min, with a flow rate of 1 mL/min at 30 °C. Injection volume was 10 µL and the metabolites were detected at 254 nm.

### 3.5. Isolation and Characterization of Compounds from X. grammica Transformant TR-74

The culture filtrate (1.0 L) of the TR-74 strain was partitioned twice with equal volumes of EtOAc. The evaporation of organic extracts yielded 1.92 g for the EtOAc layer. The EtOAc layer was separated by silica gel column chromatography (3.6 cm × 60 cm; Kiesel gel 60, 100 g, and 60–230 mesh; E. Merck, Darmstadt, Germany) with CHCl_3_/MeOH (97:3, *v*/*v*) to obtain five fractions (F1–F5). Among these, F3 was purified using a Sep-Pak C_18_ cartridge (35 cc Vac, 10 g; Waters, Milford, MA, USA) to separate two pure compounds: compounds **1** (12 mg) and **2** (10.2 mg). The fractions were monitored using TLC with the developing solvent CHCl_3_/MeOH (97:3, *v*/*v*). To determine the purity of the isolated compounds, they were subjected to HPLC (HPLC; Waters, Milford, MA, USA).

The chemical structures of the two purified compounds were determined by one- and two-dimensional nuclear magnetic resonance (NMR) spectroscopy and mass spectrometry (MS). The ^1^H- and ^13^C-NMR spectra were measured in CD_3_OD (Bruker Biospin GmbH, Rheinstetten, Germany) with an AMX-500 spectrometer (Bruker Analytische Messtechnik Gmbh, Rheinstetten, Germany). Chemical shifts given in *δ* values (ppm) are a reference to the proton and carbon-13 of the solvent at 3.31 ppm and 49.0 ppm, respectively. The ^1^H and ^13^C NMR assignments were supported by heteronuclear multiple-quantum coherence (HMQC) and heteronuclear multiple-bond correlation (HMBC) experiments; their electron impact mass spectra (EI-MS) were recorded on a double-focusing, high-resolution mass spectrophotometer (JMSDX303; JEOL Ltd., Tokyo, Japan).

### 3.6. In Vitro Nematicidal Assays

The effects of the two purified compounds (**1** and **2**) (culture filtrate) and the intermediates and derivatives of grammicin biosynthesis on *M. incognita* J2 were tested at the concentration range of 3.91–500 µg/mL. The chemicals were dissolved in methanol or acetone. Approximately 50 J2 per well were transferred to the 96-well tissue culture plates (Becton, Dickinson and Co., Franklin Lakes, NJ, USA). As negative controls, 1% methanol and acetone were used. The plates were incubated in the dark with 100% humidity at 25 °C. The experiments were repeated twice with four replicates. Observations of J2 were made under an optical microscope (Leica DM IL LED; Leica Microsystems CMS GmbH, Wetzlar, Germany), and mortality was evaluated after 3 days of treatment. Individuals were considered dead when their body did not move when probed with a fine needle [35]. Mortality rates were calculated using Abbott’s formula, [36] as follows:

$$Mortality\left(\%\right)=\frac{motality\%\;in\;treatment-mortality\%\;in\;negative\;control}{100\;-mortality\%\;in\;negative\;control}\times 100$$

*C. elegans* L4 worms were washed with a M9 buffer, and approximately 50 worms per well were transferred to the 96-well tissue culture plate containing 3.91–500 µg/mL of grammicin, compound **1**, and compound **2**. Sterile distilled water and methanol or acetone served as a negative control. The treatment plates were incubated for 3 days in the dark with 100% humidity at 20 °C [37]. The experiments were repeated twice, with four replicates. *C. elegans* mortality was calculated as mentioned above.

### 3.7. Preparation of Wettable Powder (WP)

The wettable powder formulations at 20% (WP20) were prepared as per Kim et al. (2018) [13]. Briefly, 0.8 g of 2,5-dihydroxybenzaldehyde (compound **2**) (Sigma-Aldrich, Seoul, Korea), *m*-cresol (**3**), toluquinol (**5**), grammicin (**9**), and 2,4-dihydroxybenzaldehyde (**10**) were dissolved in methanol and then separately mixed with 0.6 g synthetic hydrated silicon dioxide (white carbon; Rhodia Asia Pacific Pte. Ltd., Kallang, Singapore). This was followed by mixing with 0.2 g sodium dodecyl sulfate (CR-SDS; Yoosung Chemical R&T Co. Ltd., Daejeon, Korea), 0.2 g of sodium poly(naphthalene-formaldehyde)sulfonate (CR-WP100; Yoosung Chemical R&T Co. Ltd., Daejeon, Korea), and 2.2 g of kaoline. Sodium dodecyl sulfate and sodium poly sulfonate were used as wetting surfactant and dispersal agents, respectively. The resultant formulations were then ground in a mortar to obtain 2,5-dihydroxybenzaldehyde-WP20 (**2**-WP20), *m*-cresol-WP20 (**3**-WP20), toluquinol-WP20 (**5**-WP20), grammicin-WP20 (**9**-WP20), and 2,4-dihydroxybenzaldehyde-WP20 (**10**-WP20).

### 3.8. In Vivo Pot Experiments

The pot experiments were conducted in an incubation room (25 ± 5 °C) at Chonnam National University, South Korea. Tomato seeds (cv. Seokwang; Farm Hannong Co., Seoul, Korea) were sown in horticultural nursery soil (peat moss 14.73%, zeolite 4%, perlite 7%, vermiculite 6%, cocopeat 68%, fertilizer 0.201%, wetting agent 0.064%, and pH regulator 0.005%) and maintained for 4 weeks. Tomato seedlings (at the 4–5 leaf stage) were transplanted into 9.5 cm diameter pots containing air-dried and steam-sterilized soil (sand:horticultural nursery soil, 1:1, *v*/*v*). The WP20 test samples, at 500-fold dilution, were applied twice by soil drench (20 mL/pot): one day before inoculation and seven days after the first treatment. The final concentration of compounds in the soil was 17.78 µg/mL. The treated tomato seedlings were inoculated with 1500 *M. incognita* J2 (2 days after transplantation). Distilled water was used as a negative control and two chemical nematicides, abamectin (Terranoba^®^ containing 1.8% abamectin SL, 5000-fold dilution, and 0.002 mM; Syngenta, Seoul, Korea), and fosthiazate (Sunchungtan^®^ containing 30% fosthiazate SL, 4000-fold dilution, and 0.265 mM; Farm Hannong Co., Seoul, Korea) were used as positive controls. The pot experiments were repeated twice, with three replicates per treatment. After six weeks of nematode inoculation, to evaluate the galling index (GI), the tomato plants were uprooted and washed gently with running water to remove soil. The GI was determined as follows: 0 = 1–10, 1 = 11–20, 2 = 21–50, 3 = 51–80, 4 = 81–90, and 5 = 91–100 root galls [38].

### 3.9. Statistical Analysis

Data were evaluated by one-way ANOVA and significant differences of means were determined by Duncan’s multiple range test (*p* < 0.05) using SPSS software (v. 23.0 for Windows; SPSS, Chicago, IL, USA). The 50% lethal concentration (LC_50_) values were calculated by probit regression analysis using a linear regression model implemented in Microsoft Excel (v. 2013).

## Figures and Tables

**Table 1:** LC_50_ values for *Meloidogyne incognita* second-stage juvenile mortality subjected to various compounds, including two purified metabolites produced by the grammicin-knockout mutant strain (TR74) of *Xylaria grammica*.

Compound Number	Chemical Name	LC_50/72 h_ (µg/mL)	*R* ^2^
1	2-(Hydroxymethyl)cyclohexa-2,5-diene-1,4-dione	183	0.96
2	2,5-Dihydroxybenzaldehyde	42.43	0.97
3	*m*-Cresol	27.45	0.96
4	3-Hydroxybenzyl alcohol	27.77	0.97
5	Toluquinol	11.13	0.99
6	3-Hydroxybenzaldehyde	65.38	0.98
7	3-Hydroxybenzoic acid	>500	0.97
8	2,5-Dihydroxybenzoic acid	>500	0.86
9	Grammicin	15.95	0.96
10	2,4-Dihydroxybenzaldehyde	24.62	0.99
11	3,4-Dihydroxybenzaldehyde	75.59	0.99
12	3,5-Dihydroxybenzaldehyde	145	0.99
13	2-Hydroxybenzaldehyde	121.1	0.91
14	4-Hydroxybenzaldehyde	73.95	0.99

Each value represents the mean ± standard deviation with four replicates.

**Table 2:** Efficacy of wettable powder formulations of 2,5-dihydroxybenzaldehyde (**2**-WP20), *m*-cresol (**3**-WP20), toluquinol (**5**-WP20), grammicin (**9**-WP20), and 2,4-dihydroxybenzaldehyde (**10**-WP20) in the controlling of *Meloidogyne incognita* on tomato plants.

Tested Product	Dilution Factor	Galling Index	% Control Based on Galling Index	Fresh Root Weight (g)	Fresh Shoot Weight (g)
2-WP20	×500	3.33 ^a^ ± 0.58	8.33 ^b^ ± 14.43	4.70 ^bc^ ± 0.29	30.64 ^a^ ± 3.58
3-WP20	×500	2.67 ^a^ ± 0.58	16.67 ^b^ ± 28.87	4.42 ^bc^ ± 0.33	32.78 ^a^ ± 4.64
5-WP20	×500	1.0 ^b^ ± 1.00	72.22 ^a^ ± 25.46	4.54 ^bc^ ± 0.58	27.57 ^a^ ± 5.60
9-WP20	×500	0.67 ^b^ ± 1.16	77.78 ^a^ ± 38.49	4.56 ^bc^ ± 1.07	32.54 ^a^ ± 7.50
10-WP20	×500	2.67 ^a^ ± 0.58	16.67 ^b^ ± 28.87	4.37 ^bc^ ± 0.21	28.70 ^a^ ± 3.97
Abamectin	×5000	2.67 ^a^ ± 0.58	16.67 ^b^ ± 28.87	3.97 ^c^ ± 1.13	28.74 ^a^ ± 10.22
Fosthiazate	×4000	0.00 ^b^ ± 0.00	100.00 ^a^ ± 0.00	4.87 ^bc^ ± 0.55	29.52 ^a^ ± 3.46
Inoculated untreated control	-	3.50 ^a^ ± 0.58	-	5.69 ^ab^ ± 1.21	31.87 ^a^ ± 2.05
Non-inoculated	-	0.00 ^c^ ± 0.00	-	6.43 ^a^ ± 0.53	31.91 ^a^ ± 2.55

Each value represents the mean ± standard deviation with three replicates. Relationships among means were analysed with one-way analysis of variance and Duncan’s multiple-range test (*p <* 0.05). Means with the same letter (^a,b,c^) are not significantly different.

## Data Availability

Data is contained within the article or supplementary material.

## Supplementary Materials

The following are available online. Table S1: ^1^H- and ^13^C- NMR data for compound 1 in CD_3_OD. Table S2: ^1^H- and ^13^C-NMR data for compound 2 in CD_3_OD.

## Sample Availability

Samples of the compounds are available from the corresponding author.
