# Supplementary material for: Nematicidal Activity of Grammicin Biosynthesis Pathway Intermediates in Xylaria grammica KCTC 13121BP against Meloidogyne incognita
Source: Molecules. 2021 Aug 2;26(15):4675. doi: 10.3390/molecules26154675 (PMC8348278; doi:10.3390/molecules26154675)
Supplement: Supplementary file 1 [file molecules-26-04675-s001.zip › molecules-1313314-supplementary.pdf]

Nematicidal activity of grammicin biosynthesis pathway intermediates in *Xylaria grammica* KCTC 13121BP against *Meloidogyne incognita*

Yoon Jee Kim[†,1], Kalaiselvi Duraisamy[†,1], Min-Hye Jeong[2], Sook-Young Park[2], Soonok Kim[3], Yookyung Lee[1], VanThi Nguyen[1], Nan Hee Yu[1], Ae Ran Park[1], and Jin-Cheol Kim[1,*]

SUPPLEMENTARY MATERIAL

Table S1: ^{1}H and ^{13}C NMR data for compound **1** in CD_3OD

**Position**	δ_C	δ_H	**HMBC**
1	189.21	·	
2	150.23	·	
3	131.39	6.76	C-1, C-2, C-4, C-7
4	188.79	·	
5	137.9	4.43	C-1, C-2, C-4
6	137.49	4.43	C-1, C-2, C-4
7	59.03	4.58	C-2, C-3
OH	·	2.16	

Table S2: ^{1}H and ^{13}C NMR data for compound **2** in CD_3OD

**Position**	δ_C	δ_H	**HMBC**
1	122.71	·	
2	117.54	7.04	C-3, C-4, C-6, C-7
3	151.68	·	
4	126.25	7.03	C-2, C-3, C-6
5	119.12	6.82	C-1, C-3, C-6
6	156.03	·	
7	197.5	9.94	C-1, C-2, C-6
